# IMPLEMENTING AND EVALUATING MULTILINGUAL, COMPETENCY-BASED DEMENTIA TRAINING FOR IHSS CAREGIVERS IN CALIFORNIA

**DOI:** 10.1093/geroni/igae098.4255

**Published:** 2024-12-31

**Authors:** Jarmin Yeh, Matthew Beld, Brittney Pond, Mel Neri, Suzanna Martinez, Andrea Garcia, Moraima Castaneda, Corinne Eldridge

**Affiliations:** University of California, San Francisco, San Francisco, California, United States; University of California, San Francisco, San Francisco, California, United States; University of California, San Francisco, San Francisco, California, United States; University of California, San Francisco, San Francisco, California, United States; University of California, San Francisco, San Francisco, California, United States; Center for Caregiver Advancement, Los Angeles, California, United States; Center for Caregiver Advancement, Los Angeles, California, United States; Center for Caregiver Advancement, Los Angeles, California, United States

## Abstract

The rising prevalence of Alzheimer’s dementia in California’s aging population underscores the urgent need for a well-trained direct care workforce to help older adults age in place. This study implemented and evaluated competency-based dementia training for caregivers in California’s Medicaid-Funded In-Home Supportive Services (IHSS) program from 2019 to 2024. The program, offered in English, Spanish, Cantonese, and Mandarin, transitioned from in-person to online delivery due to COVID-19, providing an opportunity to assess this novel online approach. Using a quasi-experimental, mixed-methods design guided by the RE-AIM framework (Reach, Effectiveness, Adoption, Implementation, and Maintenance), we evaluated training outcomes, curriculum, staff roles, and delivery logistics. Of 722 caregivers who completed the training, 488 were included in the analytic sample, and 50 participated in interviews. Additionally, 11 key informant stakeholders were interviewed. Mean scores from validated instruments evaluating caregivers’ dementia knowledge (Toye et al., 2014) and self-efficacy (Fortinsky et al., 2002) showed statistically significant increases between pre-post-training and pre-3-month follow-up (p< 0.001). Thematic analysis of caregiver interviews revealed diverse pathways into caregiving, emotional rewards, enhanced advocacy skills, and improved interactions with medical providers. Stakeholder interviews uncovered implementation barriers and facilitators to intervention fidelity, and strategies for addressing the critical need for a skilled dementia direct care workforce. The training’s adaptability to online formats and multiple languages demonstrates its real-world efficacy and potential readiness for trial design. This study highlights the importance of evidence-informed, culturally and linguistically appropriate training for IHSS caregivers in managing the health and well-being of older adults living with Alzheimer’s dementia.

